# Tactile Motion Adaptation Reduces Perceived Speed but Shows No Evidence of Direction Sensitivity

**DOI:** 10.1371/journal.pone.0045438

**Published:** 2012-09-24

**Authors:** Sarah McIntyre, Alex O. Holcombe, Ingvars Birznieks, Tatjana Seizova-Cajic

**Affiliations:** 1 Faculty of Health Sciences, University of Sydney, Sydney, Australia; 2 School of Psychology, University of Sydney, Sydney, Australia; 3 School of Biomedical and Health Sciences, University of Western Sydney, Sydney, Australia; 4 School of Medical Sciences, University of New South Wales, Sydney, Australia; 5 Neuroscience Research Australia, Sydney, Australia; Bielefeld University, Germany

## Abstract

**Introduction:**

While the directionality of tactile motion processing has been studied extensively, tactile speed processing and its relationship to direction is little-researched and poorly understood. We investigated this relationship in humans using the ‘tactile speed aftereffect’ (tSAE), in which the speed of motion appears slower following prolonged exposure to a moving surface.

**Method:**

We used psychophysical methods to test whether the tSAE is direction sensitive. After adapting to a ridged moving surface with one hand, participants compared the speed of test stimuli on the adapted and unadapted hands. We varied the direction of the adapting stimulus relative to the test stimulus.

**Results:**

Perceived speed of the surface moving at 81 mms^−1^ was reduced by about 30% regardless of the direction of the adapting stimulus (when adapted in the same direction, Mean reduction = 23 mms^−1^, SD = 11; with opposite direction, Mean reduction = 26 mms^−1^, SD = 9). In addition to a large reduction in perceived speed due to adaptation, we also report that this effect is not direction sensitive.

**Conclusions:**

Tactile motion is susceptible to speed adaptation. This result complements previous reports of reliable direction aftereffects when using a dynamic test stimulus as together they describe how perception of a moving stimulus in touch depends on the immediate history of stimulation. Given that the tSAE is *not* direction sensitive, we argue that peripheral adaptation does not explain it, because primary afferents *are* direction sensitive with friction-creating stimuli like ours (thus motion in their preferred direction should result in greater adaptation, and if perceived speed were critically dependent on these afferents’ response intensity, the tSAE should be direction sensitive). The adaptation that reduces perceived speed therefore seems to be of central origin.

## Introduction

Exploratory movement of the fingers across a surface is crucial for determining the physical properties of that surface and when humans discriminate different surfaces based on their roughness, performance is better when there is relative motion between the skin and the surface [1], [2]. Furthermore, the speed of such motion can vary without affecting performance [3]. One explanation for this is that the speed is accounted for in the neural computation of surface features [4]–[6]. Little is known about how the speed of a moving surface is perceived, and this is crucial for understanding functional mechanisms of tactile perception.

We use an adaptation paradigm to study perceived speed of a moving surface, and its relationship with direction of motion. Adaptation can serve as a powerful tool to investigate the mechanisms of sensory coding of a feature of interest (see, e.g., [7]–[9]). For a long time, researchers used a simple psychophysical paradigm involving exposure to a moving surface (adaptation) followed by exposure to a stationary surface to test for an illusory motion aftereffect. They expected to find a tactile equivalent of the visual motion aftereffect (MAE; see [10]) in which the stationary test stimulus appears to move in the opposite direction to the previously adapting motion. While motion aftereffects were found, they were often inconsistent in their perceived direction, i.e., there was no reproducible *negative* (opposite direction) motion aftereffect when using the above, stationary-test paradigm [10]–[16].

More recently, studies using dynamic test stimuli (achieved with vibrating pin arrays) rather than stationary surfaces at test have found a reliable negative tactile motion aftereffect or “tMAE” [17]–[19]. This result shows that tactile motion mechanisms adapt, and is consistent with theories of motion coding, originally proposed in vision, in which the percept is determined by the difference between the activity in neurons that code opposite directions of motion. When one group of cells adapt, the ratio shifts in favour of the non-adapted cells, influencing subsequent perception of a neutral stimulus (e.g., [20], [21]). In contrast to vision, a study of direction-sensitive cells in the primary somatosensory cortex (SI) [22] found 93% were non-opponent. Opponent cells have a high resting discharge rate and get excited by one (broad) direction of motion and inhibited by another - properties that could, in principle, create tMAE in a static test stimulus. Non-opponent neurons that comprise a majority in SI would not respond as well to a static stimulus, but any dynamic test stimulus (a test stimulus involving changing stimulation of the skin over time including motion or vibration) able to excite them should reveal a population change in direction coding due to adaptation.

*Speed*, which is a feature of surface motion to which humans are perceptually sensitive [23], could in principle be coded in the same neural channels that code direction. If so, then adaptation to motion in one direction should affect perceived speed for that direction but not necessarily for the opposite. Stöber (reported in [24]) found a 24% drop in perceived speed following 4 minutes of adaptation to motion of a textured celluloid strip. We label this effect the “tactile speed aftereffect” (tSAE; our preliminary findings were reported in a conference presentation [25]). Another recent conference poster [26] also reported the tSAE. However, whether tactile speed adaptation is direction-specific has not been tested. This is the question we address.

In the standard adaptation paradigm we use, prolonged exposure to the adapting stimulus is followed by a test stimulus. Perception of the test stimulus after this adaptation is compared to the perception of it without prior adaptation. It is assumed that a) in the course of adaptation, some neurons in the sensory system respond; and b) the greater their response, the greater the adaptation in those neurons. Thus the largest aftereffects should occur if perception of the test stimulus relies on the same mechanisms as those adapted, habituated or fatigued during adaptation ([27]). Because we are testing for a *speed* aftereffect, our test stimulus is necessarily dynamic (in this case a moving surface) and should excite direction-sensitive cortical neurons, similar to previous studies that successfully reported a consistent *direction* aftereffect [17], [18]. This is an optimal stimulus for detecting any influence of direction on the tSAE.

We use a psychophysical method of constant stimuli to test whether the tSAE is direction sensitive. Rotating drums with textured surfaces created the motion our participants felt with their fingers. The use of a natural surface that stretches the skin distinguishes our study from many others [17], . A natural surface provides two cues to motion direction. The first is present in most studies and involves displacement across the skin, which stimulates the skin at successive locations. In the second cue, present in our study, friction also pulls the skin in the direction of motion, causing lateral skin stretch.

We found a substantial reduction in perceived speed following adaptation, but no evidence of direction sensitivity, with similar levels of adaptation regardless of the direction of the adapting motion. This result was strengthened by the results of a second experiment in which we used bilateral adaptation to isolate adapting motion direction as the only feature that differed between conditions. Here also, no effect of direction was found. To confirm that the adapting stimuli used in our psychophysical paradigm resulted in adaptation in sensory afferents, we measured activity in the primary afferents in two of our subjects, using microneurography, during exposure to the rotating drum. It is known that vibrotactile adaptation reduces the response of tactile afferents [35], [36], and although prolonged motion is likely to have a similar effect, this has not been established.

## Materials and Methods

### Participants

Nine participants volunteered, six naïve observers and three authors (2 left-handed). Written informed consent was obtained and the Human Research Ethics Committee of the University of Sydney approved the study, which was conducted according to the principles expressed in the Declaration of Helsinki.

### Apparatus

**Figure 1:**
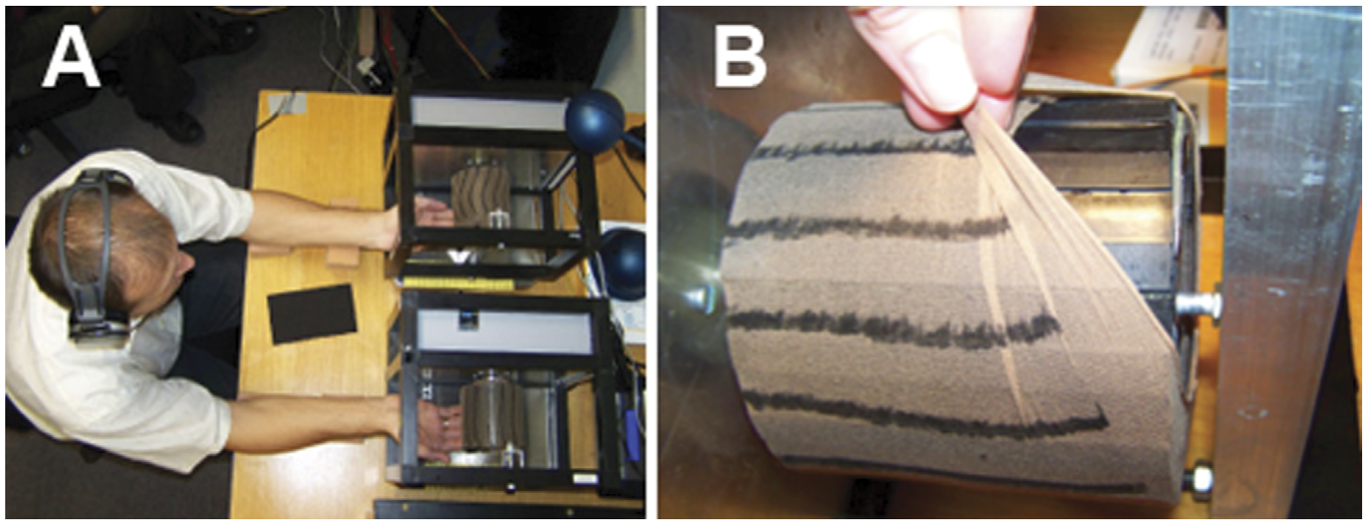
The apparatus. A: Observers judged the relative speed of two different moving drums. (The subject of the photograph has given written informed consent to publication of their photograph.) B: One of the drums used to create tactile motion. Here, the stocking is pulled back to reveal the ridged rubber surface underneath.

A hard rubber surface was attached to two cylindrical drums. The surface was textured with ridges 1 mm high and 10 mm wide, and troughs 12 mm wide. The ridges were spaced at regular intervals with a centre-to-centre distance (spatial period) of 22 mm. The surface was covered (Figure 1B) with ladies’ stocking fabric (98% nylon, 2% elastine) to reduce friction with the fingers while the drums were in motion (without the covering, prolonged exposure to the moving drum was uncomfortable). The circumference of the drums was 338 mm, and they were rotated by a stepper motor (Lineartec MOT-122 High Torque Hybrid Stepping Motor) with a step size of 1.8 degrees controlled by LabView software (National Instruments, USA). At all speeds used (see Procedure section, below), motion felt smooth and no vibration was detected from the stepper motor. Depending on the speed, there were between 8 and 72 steps of the motor per second.

### Procedure

Perceived surface speed was measured with a two-alternative forced-choice procedure involving two rotating drums. Participants rested their arms on foam cushions and gently touched the two drums (one with each hand) from underneath (Figure 1A). They were instructed to touch the drum with only the distal segments of their index, middle and ring fingers. Pigment powder on the drums resulted in marking of the areas of skin the drum contacted, and inspection of the fingers after the experiment allowed the experimenter to check that participants followed the instructions regarding finger contact area with the drums. Participants placed their fingers on the surface at the start of each run while the drums were stationary and stayed in this position for the duration of the run. Participants were instructed to close their eyes to avoid visual cues to the motion of the drums. White noise delivered through headphones masked any auditory cues.

In the test phase (preceded by an adaptation phase in some experimental conditions), both drums moved simultaneously and in the same direction for one second. Participants were asked to compare the speeds of the two drums, saying, “left” or “right” to indicate which one moved faster, and the experimenter recorded each response using a button box. The two stimuli to be compared were presented simultaneously with synchronous onset and offset. No participant ever reported that one stimulus was felt to last longer than another, and this perceived simultaneity suggests that relative duration was not used to make the judgment. One hand, the reference hand, was presented with the standard stimulus, which was the same speed on every trial. This is also the hand that was adapted in the adaptation conditions. The other hand, the comparison hand, was presented with comparison stimuli of a variety of speeds (14–122 mms^−1^). For three of the participants the right hand was the reference hand and the left was the comparison hand; for the remaining participants the reverse was true. Using the method of constant stimuli, we estimated the point of subjective equality (PSE) between the speed felt on the reference (adapted) hand and the comparison hand. The PSE indicates the perceived speed of the standard stimulus. The speed of the standard and adapting stimuli was 81 mms^−1^, a speed well within the functional range used in active surface exploration [37].

The metal frame of the drums rested on electronic scales, which measured normal (upward) contact force that participants applied to the drums with their fingers. Participants made no contact with any other part of the apparatus or the scales. Auditory tones delivered through headphones indicated to participants when they pressed too hard (with a force greater than 100 gm-wgt) or too softly (less than 10 gm-wgt) and they were asked to keep within this range throughout the experiment.

### Experiment 1

The goal of Experiment 1 was to determine whether the tSAE is direction sensitive – whether the relative direction of the adapting motion has an effect on the perceived speed of the test stimulus. Participants adapted to sustained tactile motion in different directions, then judged the speed of a subsequent tactile motion stimulus that was either the same or the opposite direction as the previously exposed adapting stimulus.

#### Design and procedure

**Figure 2:**
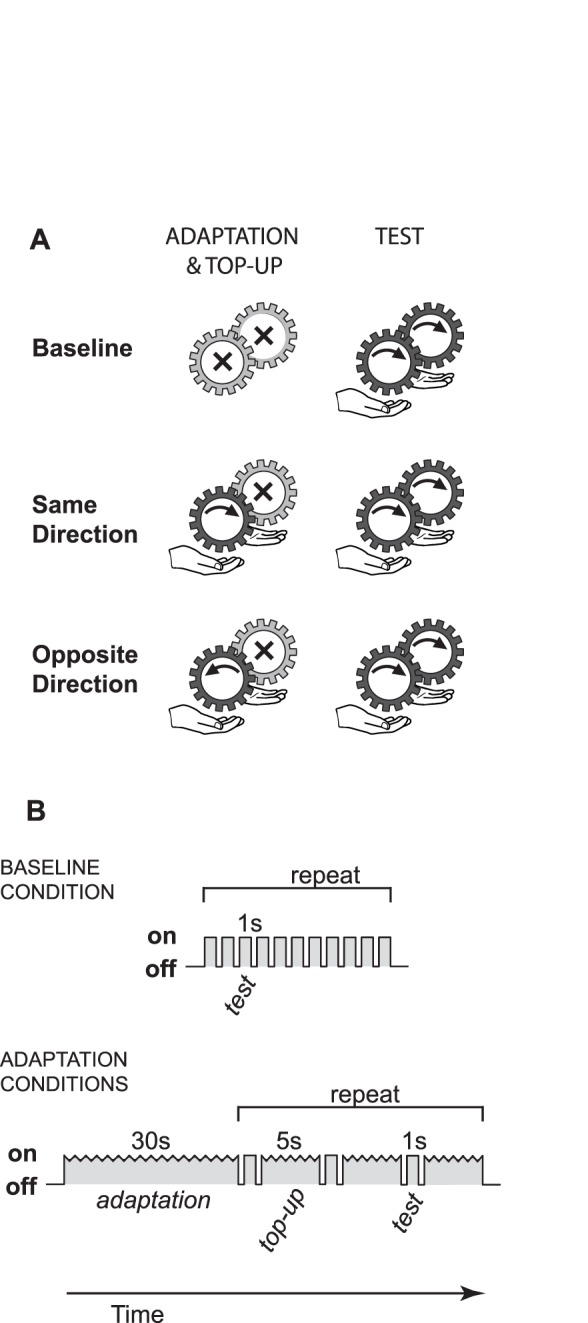
Design of Experiment 1. A: The three experimental conditions in Experiment 1. B: The stimulus sequence in the two adaptation conditions of Experiment 1.

Experiment 1 comprised three conditions: two adaptation conditions and a baseline condition (Figure2A). The adaptation conditions differed in the relative direction of the adapting stimulus motion. In the Same Direction condition, the adapting stimulus moved in the same direction as the standard and comparison stimuli (distal to proximal for three of the participants and vice versa for the remaining six). In the Opposite Direction condition, it moved in the opposite direction. During the adaptation phase, the reference hand was exposed to 30 seconds of motion (the adapting stimulus) immediately before the speed judgments began. During the test phase, the adaptation conditions also included 5 seconds of “top-up” adaptation following each left/right judgment. This stimulus sequence is illustrated in Figure 2B. In the Baseline condition, no adaptation preceded the speed judgments, and there was no top-up period during the test phase.

All participants completed the three experimental sessions (one for each condition), with the first session preceded by a short practice to familiarise participants with the task. Each session consisted of three runs, separated by two-minute rest breaks. Each session comprised 162 judgements of 9 comparison speeds (18 judgements per comparison speed). To minimise lingering adaptation effects, all sessions were preceded by a break of at least one hour following any adaptation session conducted earlier that day.

The following factors varied across participants: (1) which hand was presented with the test stimulus (left vs. right), (2) the direction of the test and comparison stimuli (proximal vs. distal), (3) the order of the two adaptation conditions (same direction first vs. opposite direction first). The nine participants were assigned values of these three factors in a pseudo-random fashion.

#### Neurophysiology

To confirm that there was adaptation at the periphery, we include microneurographic recordings obtained in two participants (SM and TSC). We recorded from primary afferents during tactile motion to document the adaptation. We obtained one single-unit recording of a type 1 fast adapting unit (FA1) and one multi-unit recording. The multi-unit recording was made while one hand was stimulated in a fashion similar to the reference (adapted) hand in the Same Direction condition of the psychophysics protocol described above. The comparison hand was not stimulated, and the participant made no responses. The single-unit recording was made from the adapted hand during an altered version of the psychophysics protocol for the Opposite Direction condition in which there were 12 presentations (instead of 18) for each comparison speed. For both the multi-unit and single-unit recordings, the fingers did not touch the stimulus until immediately prior to the start of adaptation.

The median nerve was located at the wrist by palpation and electrical stimulation via a surface probe. A tungsten microelectrode (Frederick Haer & Co. Inc., Brunswick, ME, USA) was then inserted percutaneously and guided towards the median nerve by weak electrical stimulation through the electrode tip. Once in the nerve, multiunit recordings were obtained by positioning the electrode within the fascicle innervating mechanoreceptors in the fingertip skin. Fine adjustments of electrode position were guided by auditory feedback of the neural activity associated with mechanical stimulation to the fingertip skin applied by the electrophysiologist (author IB).

Neural activity was amplified (gain 1 x10^4^) and band-pass filtered at 0.3–2.0 kHz, 50 Hz notch (Neuro Amp EX, ADInstruments, Bella Vista, Australia). All electrophysiological data were recorded and analysed on a computer-based data acquisition system LabChart 7/PowerLab (ADInstruments). Single nerve impulses (spikes) were identified by template matching and counted using the Spike Histogram module. For the multi-unit recording, no attempt was made to separate or identify individual afferents.

The participants provided informed written consent to the procedure, which was approved by the human ethics committee of the University of New South Wales and conformed to the Declaration of Helsinki.

### Experiment 2

In Experiment 1 the receptors of the adapted hand were stimulated to a greater extent overall than the non-adapted hand, and participants occasionally commented that the moving stimuli evoked different sensations in the two hands. Conceivably, this difference may have impaired comparison of the speeds felt by the two hands, obscuring any effect of direction. In Experiment 2, we eliminated all differences in stimulation between the hands except for the direction of the adapting motion. We adapted both hands, one in each direction, and measured the resulting speed adaptation using one hand as the reference and the other as comparison.

**Figure 3:**
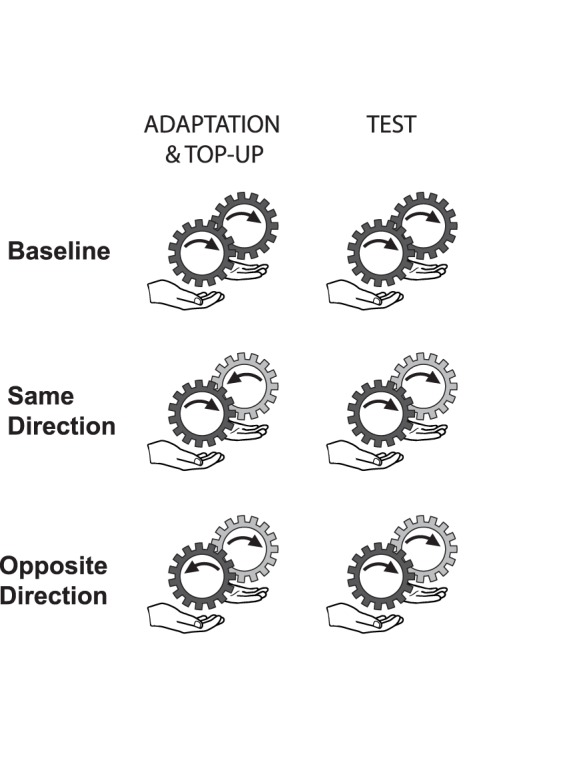
Design of Experiment 2. The three experimental conditions of Experiment 2.

The methods were the same as for Experiment 1, except as noted below. In all three conditions illustrated in Figure 3, both the reference and the standard hand were adapted with 30 seconds of continuous motion in the adaptation phase, plus the 5 seconds “top-up” adaptation following each left/right judgment during the test phase. As in Experiment 1, the test phase immediately followed the adaptation phase. The conditions of Experiment 2 differed in the direction of adaptation that was applied to the two hands. In two conditions, the adapting stimulus applied to one hand was in the opposite direction to the adapting stimulus applied to the other hand. In one of these, called the Same Direction condition, the adaptation applied to the reference hand was the same direction as that of the stimuli presented during the test phase (to both hands). In the other, Opposite Direction condition, the adaptation applied to the reference hand was in the opposite direction to the stimuli presented during the test phase. In a third condition, Baseline, the two adapting stimuli applied to the two hands were both in the same direction.

All participants completed three experimental sessions (one for each condition). The second and third sessions followed a break of at least one hour to minimise carry-over of adaptation from the previous session. The order of the conditions was randomly determined.

Unlike in Experiment 1, the PSE in Experiment 2 is not necessarily expected to be different in Baseline compared to the other two conditions because both hands were adapted. Differences should only appear if direction influenced speed adaptation. Table 1 gives the predicted outcomes of Experiment 2 given the various possible effects of direction of adaptation on perceived speed.

**Table 1:** Possible outcomes of Experiment 2.

Effect of Adaptation	PSE by Condition
Matched Direction > Opposite Direction	Opposite Direction > Baseline > Same Direction
Opposite Direction > Matched Direction	Same Direction > Baseline > Opposite Direction
Matched Direction = Opposite Direction	no differences across conditions

The first possibility is that matching the direction of the adapting and test stimuli leads to greater adaptation of perceived speed (first row). If this is the case, then the Opposite Direction condition should produce the highest PSE (smallest tSAE) and the Same Direction condition should produce the lowest PSE (greatest tSAE). This is because in the Same Direction condition, the direction of motion for the reference hand is matched at adaptation and test, whereas in the Opposite Direction condition, the motion for the reference hand at adaptation and test are in opposite directions. The second possibility is that the greatest adaptation occurs with an adapting stimulus moving in the opposite direction to the test (second row). In this case the Same Direction condition should produce the highest PSE (smallest tSAE) and the Opposite Direction condition should produce the lowest PSE (greatest tSAE). The third possibility is that the tSAE is insensitive to direction, with the adapting stimulus creating similar levels of adaptation regardless of its direction relative to test (third row). In this case, we would not expect to see any consistent differences across conditions.

### Experiment 3

Because of previously reported difficulties eliciting a negative tMAE with moving surfaces like ours [10]–[16], a reviewer questioned the capacity of these surfaces to evoke a clear enough perception of direction to ensure it provides a good test for the presence of a direction-sensitive tSAE. Experiment 3 shows that the moving surfaces we used produce a clear direction percept.

#### Participants

Six participants volunteered, three naïve observers and three authors (2 left-handed). Written informed consent was obtained and the Human Research Ethics Committee of the University of Sydney approved the study, which was conducted according to the principles expressed in the Declaration of Helsinki.

### Procedure

Using the same apparatus as in Experiments 1 and 2, participants felt the moving surface for four minutes with the index and middle fingers of their right hand. Direction of motion was distal. Participants responded to the stimulus by continuously reporting the perceived direction of motion by pressing one of three buttons: 1) distal, if the stimulus appeared to move away from their body, 2) proximal if it appeared to move towards their body, or 3) unclear, if they could not judge the direction of motion. Participants were instructed to continuously monitor their perception and press the appropriate button as soon as the direction of motion appeared to change. They were instructed to respond every few seconds even if perceived direction did not change. Participants were told that sometimes people experience illusory perceptions of motion and that they should report what they felt, rather than what they thought the stimulus was actually like. Participants were also warned that their perception might change so rapidly that their button presses could not keep up. If they experienced this, they were to report all perceived directions even if their responses lagged behind. To test for the presence of a tMAE, participants also continued reporting the perceived direction for three seconds after the drum stopped moving. Three speeds were tested, 27, 54 and 108 mms^−1^, in sessions separated by breaks of at least 2 minutes.

### Data Analysis

For the psychophysical data in Experiments 1 and 2, the proportion of responses for which the comparison stimulus was judged faster was calculated for each comparison speed. Using the statistical software R [38] with the ‘modelfree’ package [39], the data for each participant for each condition were fitted by logistic regression function. The resulting psychometric function provided the point of subjective equality (PSE), the speed for which participants were equally likely to say that the comparison was faster or slower than the standard. The PSE indicates the perceived speed of the standard stimulus. The slope of the function provides a measure of discrimination sensitivity.

For the microneurography data, the stimulus event time – that is, the timing of spikes relative to the temporal period of the ridges – was estimated via ad-hoc observation of the spike data. For each period of motion - 30 s adaptation, 1 s test, 5 s top-up - the stimulus was manually re-aligned with the spike train to account for slight variations in acceleration and deceleration time at the onset and offset of drum motion.

For the direction judgement data collected in Experiment 3, perceptual state was interpolated at 1 s intervals from button presses, then the proportion of each perceptual state (veridical direction, opposite direction, unclear) was calculated for 10 s bins.

**Figure 4:**
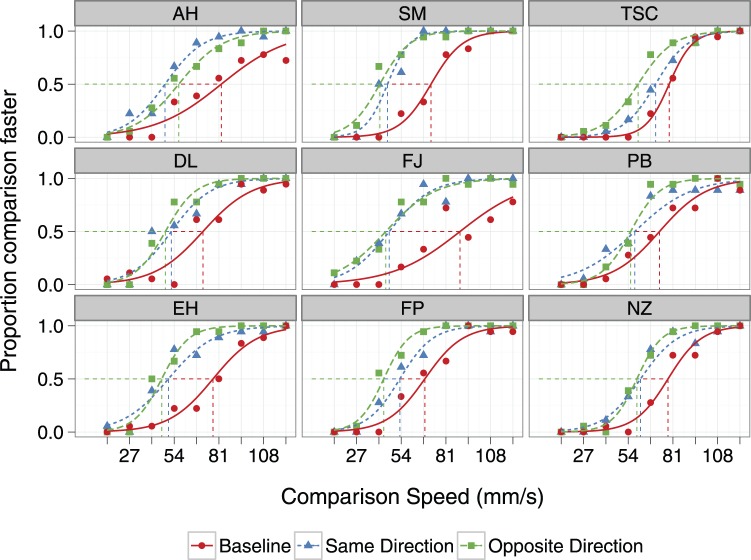
Individual psychometric functions for three experimental conditions, Experiment 1. The actual speed of the standard stimulus was always 81 mms^−1^. Comparison speed is on the abscissa. The ordinate gives the proportion of responses in which the comparison stimulus was judged faster than the test stimulus. The three experimental conditions are: no adaptation (circles), adaptation in the same direction as the test speed (triangles), and adaptation in the opposite direction to the test speed (squares). The lines are the fitted logistic regression curves. Also shown are the PSEs given by the mean of the fitted logistic function. PSEs in the baseline condition were higher in all participants (perceived speed faster) than PSEs in the adaptation conditions.

**Figure 5:**
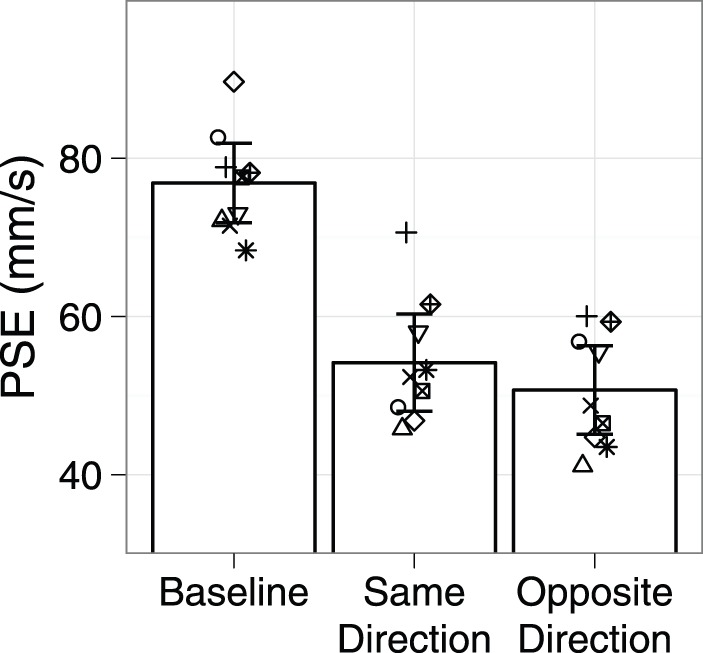
Mean PSE (bars) across participants as a function of experimental condition, Experiment 1 . Error bars represent 95% confidence intervals of the mean PSE. The symbols indicate the PSE for each participant.

**Figure 6:**
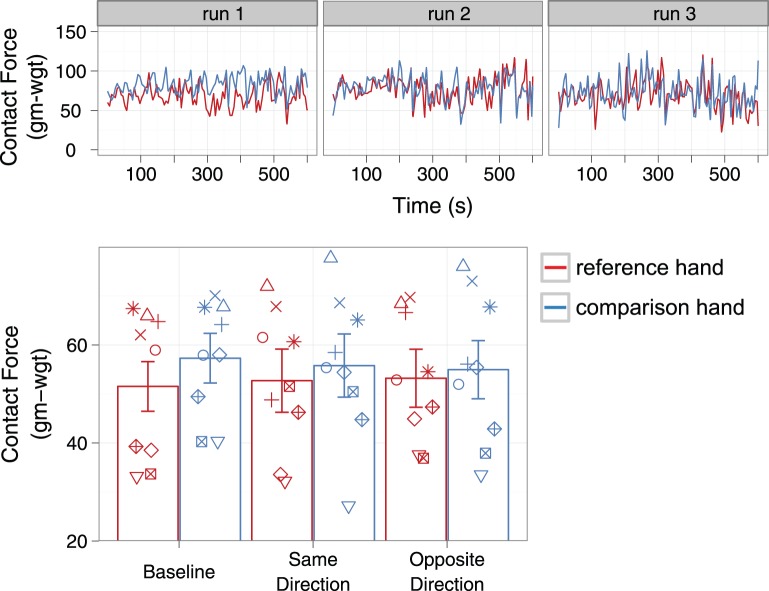
Contact Force data. A: Contact force over time for the reference (black) and comparison (white) hands for one representative participant (DL) in the Same Direction condition. The session consisted of three successive runs, represented by one panel each. B: Mean contact force for the reference and comparison hands for each condition. Error bars on each pair of bars within the same condition are identical and represent 95% CI of their difference scores (comparison – reference).

Figures 4–[6] and [8]–[11] were generated using the R package “ggplot2” [40]. Data and statistical scripts are available at The Sydney eScholarship Repository (http://ses.library.usyd.edu.au/handle/2123/8648).

## Results

### Experiment 1

#### Perceived speed

The results of Experiment 1 revealed a substantial tSAE in all participants – adaptation reduced perceived speed. Compared to Baseline, adapting the hand decreased perceived speed by an average of 30%. Psychometric functions are shown in Figure 4. The direction of the effect is the same for all nine participants: the curves for the Same Direction and Opposite Direction adaptation conditions are shifted to the left of the curve for the Baseline condition, indicating that perceived speed in the adaptation conditions was lower.

The mean PSE across participants is shown in Figure 5. When adapted in the same direction as the test, the mean PSE was reduced 23 mms^−1^ (SD = 11) from Baseline; following adaptation in the opposite direction, the mean reduction in PSE was 26 mms^−1^ (SD = 9). A repeated measures ANOVA shows a significant effect of condition (F_2,16_ = 49.20, p<.001). Post-hoc contrasts revealed a significant difference between mean Baseline PSE and those in the two adaptation conditions (p<.001). The direction of the adapting stimulus relative to the test stimulus did not matter; the PSEs of the two adaptation conditions did not significantly differ (mean difference = 3 mm/s, p = .09).

The slope of the psychometric function is a measure of discrimination sensitivity. There were no significant differences in slope between the conditions, (Baseline mean slope = 0.021, SD = 0.007; Same Direction mean slope = 0.023, SD = 0.006; Opposite Direction mean slope = 0.027, SD = 0.006), the difference was not significant (F_2,16_ = 2.91, p = .08). This stands in contrast to the subjective experience of participants, several of whom spontaneously reported that the ridges of the moving drum felt less clear after prolonged exposure. Following adaptation runs, they reported some “numbness” when touching surfaces and objects, which faded over time. However, this subjective numbness did not influence speed discrimination performance.

#### Contact force

The contact force data show that the participants successfully exerted similar contact force on both drums across all conditions, and indicate that changes in contact force over the course of the experiment cannot account for any adaptation effects. Figure 6A shows the normal (upward) contact force that was applied by one representative participant (DL) during the Same Direction adaptation session. The session comprises three runs (depicted in three panels), each starting with 30 s adaptation, followed by fifty-four 1 s comparisons and fifty-four 5 s top-ups. Figure 6B gives the mean normal contact force across all subjects and conditions.

The overall mean contact force was 52 gm-wgt (SD = 13) applied by the reference hand, and 56 gm-wgt (SD = 13) applied by the comparison hand. The mean contact force applied by the two hands for the Baseline, Same Direction and Opposite Direction conditions was 54 (13), 54 (14) and 54gm-wgt (14) respectively. A repeated measures ANOVA revealed no significant main effect of hand (F_1,8_ = 3.36, p = .10) nor condition (F_2,16_ = 0.01, p = .99), nor was there a significant interaction effect between hand and condition (F_2,16_ = 1.03 p = .38).

#### Neurophysiological data

**Figure 7:**
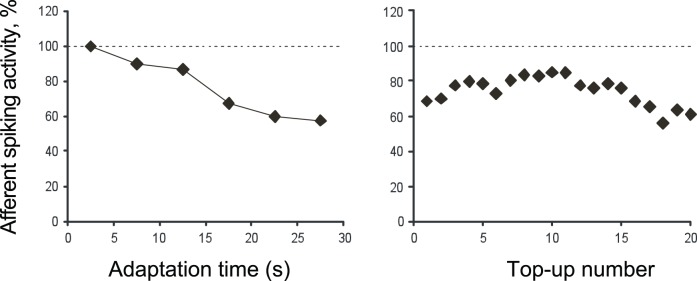
Multiunit recording of neural activity in primary afferents exposed to prolonged tactile motion. A. Relative changes in the spiking activity during 30 s long adaptation phase. Data averaged over 5 s time bins. B. Level of spiking activity during twenty 5 s long top-up periods in the test phase, relative to the same baseline as in A.

It was expected that peripheral afferents would show reduced activity following exposure to sustained motion stimulation. Our microneurographic recordings illustrate this adaptation. Both the multiunit recording and the single FA1 recording showed a decrease in unit activity over time in response to prolonged stimulation, indicating that significant primary afferent adaptation took place. After 30 s adaptation to the 81 mms^−1^ adapting motion, the multi-unit recording had decreased to 57% of its initial level of 33.4 impulses per second (ips; see Figure 7). For the single-unit (FA1) recordings, activity was recorded during two runs of the stimulus protocol used in the Opposite Direction condition (see Materials and Methods section for details), and the recording was successful for run 2 only (there was a two-minute rest between the runs). During run 2, the firing rate of the FA1 was initially 6.8ips during the adaptation phase and continued at 8.3ips for the next 30 s of top-up adaptation. In the last 30 s of top-ups, after approximately 4 minutes of adapting motion, the firing rate had reduced to 3.4ips, 50% of its initial level.

**Figure 8:**
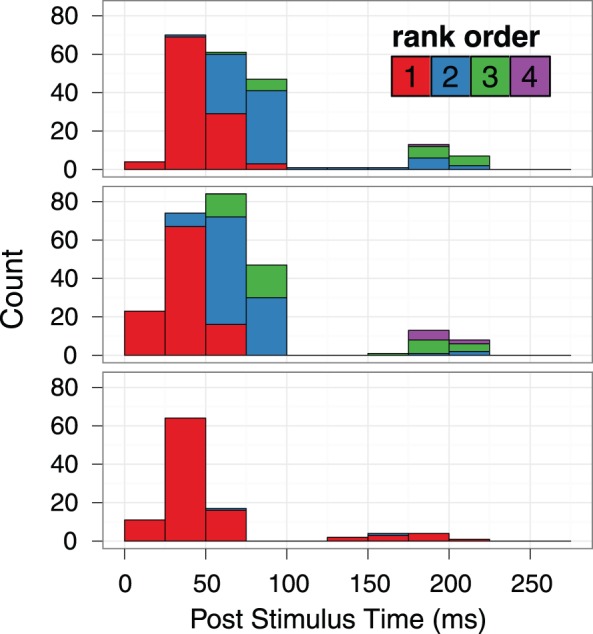
Frequency histograms showing the timing of spikes relative to surface ridges (278 ms temporal period). The stimulus event time was recovered via ad-hoc observation of the spike data. Three key stages of run 2 are shown: 1) Top panel: the 30 s adaptation period; 2) middle panel: the first five top-ups; and 3) bottom panel: the last five top-ups. Shades give the rank order of the spikes after the onset of a given ridge.

For a more detailed picture of how adaptation affected the response of the FA1 unit, we examined the temporal profile of its response at key stages of run 2. Figure 8 is a post stimulus spike histogram, showing the timing of the spikes within a temporal window equal to the period of the surface profile (278 ms). The three key stages of run 2 shown are 1) the 30 s adaptation period (continuous proximal motion) at the start of the run; 2) the first six top-ups (30 s proximal motion stimulation time) immediately following adaptation; and 3) the last six top-ups (30 s proximal motion stimulation time) at the end of the run. Over 4 minutes of motion stimulation occurred between the first six and the last six top-up periods.

The top panel of Figure 8 shows the adaptation period of run 2. Nearly every time (98%) a ridge moved over its receptive field, the afferent responded faithfully, with the first spike precisely phase-locked to the ridge movement. These initial spikes were often followed by a few more: multiple spikes were evoked by a ridge for 76% of the 107 ridge presentations during this period. A similar pattern is also present in the middle panel, which shows the first six top-up periods following adaptation. Again, the afferent responded to nearly all (98%) of the 108 ridge presentations, and 89% of these generated multiple spikes. A pattern consistent with adaptation is evident in the bottom panel, which shows the last six top-up periods of the run. Here, 84% of the 108 ridges evoked a spike that was precisely time-locked to the ridge onset, while only two ridge presentations (1.9%) evoked multiple spikes.

The results indicate that the steps of the motor driving the surface rotation did not cause vibration that stimulated primary afferents. For the adapting and standard speed of 80 mms^−1^, the motor stepped approx. 50 times per second, whereas the ridges of the surface passed over the skin about 4 times per second. The single unit recording was precisely phase-locked to the timing of the ridges (see Figure 8), indicating that the ridges rather than the steps of the motor drove the neural response. The maximum firing rate of the two recordings (33.4ips for the multi-unit recording, 6.8ips for the single-unit) never approached the frequency of the motor steps (50 Hz). Therefore, if the stepper motor generated any vibration, it was below the threshold of primary afferents from which we recorded.

Because we applied the Opposite Direction stimulus protocol, in which different directions of test and top-up periods alternated, we were able to determine the FA1 unit’s direction preference. We did this by comparing its response during the 1 s test periods, in which the drum moved in a distal direction, with its response in the first 1 s of the 5 s top-up periods, when it moved in a proximal direction. We used the first six top-up and test periods, before the unit had shown a substantial reduction in response due to adaptation. During this period the FA1 displayed a preference for proximal motion, responding at a rate of 9.3ips, compared to 4.2ips for distal motion.

### Experiment 2

**Figure 9:**
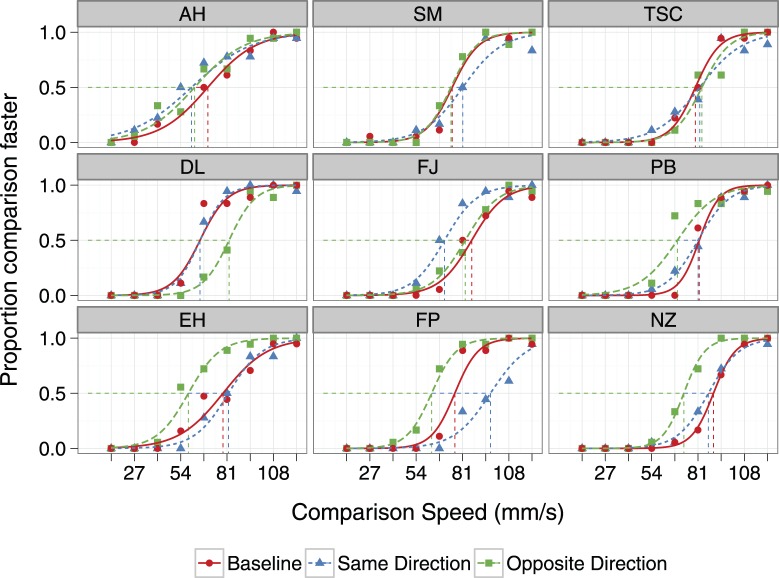
Individual psychometric functions for three experimental conditions, Experiment 2. Format the same as in Figure 4. Data are plotted for three adaptation conditions: baseline (circles), in which both hands received adaptation in the same direction; same direction (triangles), in which the reference hand was adapted in the same direction as test, and the comparison hand was adapted in the opposite direction; and opposite direction (squares), in which the reference hand was adapted in the opposite direction to test, and the comparison hand was adapted in the same direction.

The results of Experiment 2 revealed no significant differences between conditions – the effect of adaptation did not significantly depend on its direction relative to the test. Figure 9 shows the psychometric functions for the three conditions for each observer. The response curves for the same direction and opposite direction conditions are not shifted in any consistent direction relative to baseline. The non-significant shifts that are sometimes visible are usually small, with considerable overlap of the response curves for the different conditions.

**Figure 10:**
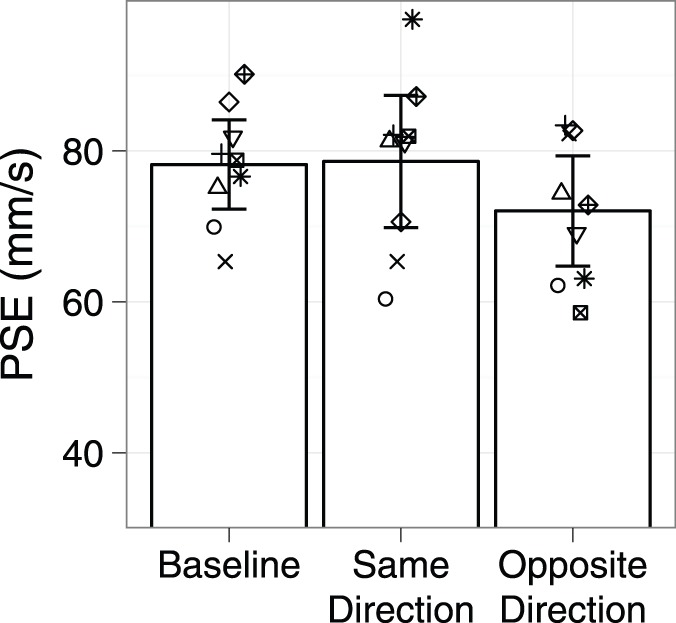
Mean PSE (bars) across participants as a function of adaptation condition, Experiment 2. Error bars represent 95% confidence intervals of the mean. The symbols indicate the PSE obtained for each participant.

The PSEs across participants are given in Figure 10. The mean PSE was 78 mms^−1^ (SD = 8) in Baseline, 79 (SD = 11) in the Same Direction condition and 72 (SD = 9) in the Opposite Direction condition. These small differences were not statistically significant according to a repeated measures ANOVA (F_2,16_ = 1.42, p = .27).

We also examined whether discrimination sensitivity was affected by adaptation direction, using slope of the logistic regression as an index of discrimination sensitivity. There was a trend for a slightly steeper slope in the Baseline condition, although the effect of condition did not reach significance with a repeated measures ANOVA (F_2,16_ = 3.62, p = 0.05). The mean slope was 0.031 (SD = 0.008) in Baseline, 0.025 (0.006) in the Same Direction condition and 0.029 (0.007) in the Opposite Direction condition.

### Experiment 3

**Figure 11:**
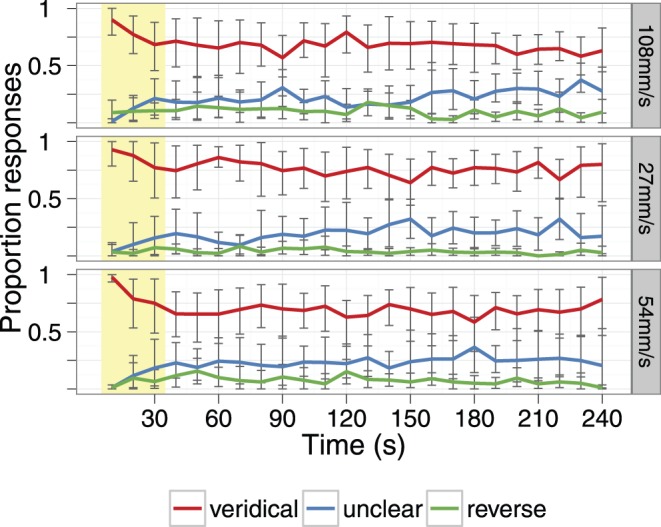
Perceived direction of the moving surface over time. The proportion of time each response was given is plotted over a four-minute period. Data are averaged over 10 s time bins. Error bars give the bootstrapped 95% confidence interval of the mean.

Out of three possible responses regarding direction of motion in this experiment - veridical, reverse and unclear – the most frequent direction perceived was veridical throughout the 4 minutes of the run. Figure 11 shows the mean probability of each response for 6 participants. The mean probability of the veridical response averaged across runs was.77,.70 and.68 for the 27, 54 and 108 mms^−1^ speeds, respectively. The mean probabilities of the veridical response within the first 30 s – equivalent to the duration of our adaptation period in Experiments 1 and 2– was even higher:.86,.84 and.79 for the 27, 54 and 108 mms^−1^ speeds, respectively.

After the drum stopped, participants continued reporting the perceived direction of the stationary surface for three seconds. Five of the six participants reported an aftereffect on at least one trial (TSC never experienced an aftereffect). Overall, a negative tMAE (illusory motion perceived in the opposite direction to the adapting stimulus) was reported in 19 of the 70 trials (27%), and a positive tMAE (in the same direction as the adapting stimulus) was reported in 4 of the 70 trials (6%). This result is consistent with previous studies that have found a low incidence of the tMAE, and that it occurs in both positive and negative directions [10]–[16].

## Discussion

We found that adapting to a surface moving across the skin reduces its perceived speed, an effect we labelled the tactile speed aftereffect (tSAE). This is the first known replication of this effect since it was reported in 1960 [24]. We also report, for the first time, that this effect is *not* direction sensitive. Experiment 1 showed that the perceived speed of the test stimulus decreases by approximately one third and does not depend on whether the adapting stimulus moved in the same or the opposite direction as the test. This insensitivity to direction was confirmed in Experiment 2 in which bilateral adaptation to motion in different directions revealed no perceptual differences. Experiment 3 demonstrated that the direction of the motion of our stimulus is clearly perceived most of the time, even during prolonged stimulation, indicating that this stimulus engages directional processes. This combined with the fact that we used a dynamic test stimulus to engage the same processes during both adaptation and test phases indicates that our stimulus is suitable for testing direction sensitivity of motion adaptation.

Our participants were asked to judge speed, but it is possible that their speed judgments were based on stimulus features other than the speed. One candidate is temporal frequency (TF), or the number of prominent ridges on our surface (see Figure 1B) stimulating the skin per unit of time. This was confounded with speed because we always used the same surface (22 mm spatial period), and with increased speed, more ridges would have touched the fingertip in the same time period (3.7 Hz for the standard stimulus, 0.6–5.5 Hz for the comparison stimuli). This confound could in principle explain our findings if our participants relied on the TF of the ridges, and if perceived TF is susceptible to adaptation. We think it is unlikely that our participants based their judgments on TF, primarily because people are able to judge the speed of the moving surface independently of its TF [23]. If relying on TF offered some advantage, it might be a preferred strategy, but it is not obvious what advantage it would have offered to our participants.

It is also unlikely that adaptation of perceived temporal frequency can explain our results. Our microneurography data from the present study suggest that information about the TF of the stimulus was preserved following adaptation. Adaptation to the duration of the interval between successive ridges hitting the skin could also have occurred (274 ms for the standard stimulus, 183–1644 ms for the comparison stimuli). Tactile interval durations are subject to adaptation, making them appear shorter [41]. However, if this occurred in our experiment perceived speed should increase, which is the opposite of what we observed.

Furthermore, preliminary results of another study in our laboratory, in which we completely dissociated speed from either TF or interval duration by using a number of different speeds and surfaces, suggest strongly that speed rather than TF or duration explains the adaptation effects, and this in turn supports our proposal that tSAE is based on speed judgments, rather than on judgments of other features. This preliminary result also suggests that the adaptation responsible for the shift in speed judgments occurs in neural channels encoding speed itself, rather than in those encoding the temporal frequency of the stimulus that feed into the speed channels.

Our results show *no* effect of direction on speed adaptation (regardless of whether the adapting motion direction was distal or proximal, the decrease in perceived speed of the test stimulus was very similar). One could argue that the result is due to a ceiling effect. If too much adaptation occurred, it would drive all primary afferents and other stimulated neurons into an equally unresponsive state, despite our intention to adapt units preferring one direction more than those preferring the opposite direction. But this scenario is unlikely. Neurophysiological studies on adaptation to vibration [35], [42] show that adaptation to a non-preferred stimulus will cause a primary afferent unit to reduce its response rate until it reaches a stable level of adaptation. However, a preferred vibration frequency or amplitude is able to create an even stronger adaptation. Because primary afferents are also sensitive to direction [43]–[47], a preferred direction of motion should also create stronger adaptation than a non-preferred direction, even after prolonged adaptation.

Since direction of motion was of no consequence to the size of the tSAE, it follows that *peripheral adaptation is not the cause of the reduction in perceived speed*. The level of adaptation in peripheral units caused by the rotating drum would vary depending on their direction preference and movement direction. An example of such a unit, preferring proximal over distal motion, is provided in our microneurography data. If adaptation of primary afferents were responsible for the tSAE, one would expect the effect to be stronger in the direction of the adapting stimulus, which is not what we found. The lack of direction sensitivity thus indicates that the adaptation that reduces perceived speed occurs centrally. A possible central mechanism that is robust to peripheral firing rates is one based on sequential activation of afferents with receptive fields positioned along the trajectory of the moving stimulus [48], [49]. Speed could be estimated from the distance between successive positions and the time between stimulation [48].

The absence of direction sensitivity of the tSAE also suggests that *speed is coded separately from direction*, i.e., in different neurons. With joint coding, where single neurons show a preference for both a particular stimulus speed and direction, we would expect a reduced response to the adapted combination of speed and direction, which we did not observe. An exception to this would be a ‘gain control’ mechanism, similar to that observed in the visual motion system of flies [50], in which activation of direction sensitive neurons transfers adaptation to units tuned to all directions. In touch, neurophysiological evidence is mixed. In support of joint coding, a subset of direction selective neurons in areas 3b and 1 show a stronger direction preference with increased speed [51], [52]. Romo and colleagues found neurons in the supplementary motor area involved in a tactile speed discrimination task, but they did not test for direction sensitivity. Support for separate coding is found in clinical evidence. Essick and colleagues [53] described patients with cortical damage whose capacity to discriminate the direction of tactile motion was either eliminated or severely impaired, while capacity to judge speed was preserved [53]. A similar though less pronounced dissociation was also reported in patients with dorsal column damage [54].

The tSAE is a perceptual aftereffect of adaptation to tactile motion. A reliable aftereffect also occurs for perception of motion direction [17]–[19]. The use of a *dynamic* test stimulus is a characteristic of our speed adaptation study that is shared with “successful” tMAE studies that found a consistent negative aftereffect in perceived direction. Watanabe and colleagues, authors of the first tMAE study that used a dynamic test [17], emphasised the importance of a good match between the adapting and the test stimuli (pp 578, 581), contrasting their experimental design with earlier studies that used a stationary test stimulus and failed to observe a reliable tMAE [11], [13], [14]. Stöber’s early investigation of the tSAE (reported in [24]) also shares this characteristic - the test stimulus was in motion (i.e., dynamic) – and similar to what we found, reported a robust and large aftereffect. In summary, the use of a stationary test produces no reliable aftereffects in touch, but the use of a dynamic one does. This contrasts with vision where both stationary and dynamic tests result in robust directional aftereffects (for review, see [55]). The full implications of the difference in response to a stationary test stimulus between vision and touch are not yet clear, but there is no doubt that tactile motion mechanisms also adapt, affecting both perceived direction and speed. Further, we can rule out the possibility that surface motion across the skin is not a good stimulus to study aftereffects. Our Experiment 3 shows that motion direction of a surface moving across the skin is clearly perceived most of the time, even after minutes of continuous stimulation.

In conclusion, our results are consistent with other tactile adaptation studies that relied on a dynamic – rather than stationary - test stimulus for robust aftereffects. We documented that the tactile speed aftereffect (tSAE) was similar in size regardless of whether the direction of the adapting and test stimuli match. Our results suggest that speed-encoding processes are robust to reductions in the firing rates of primary afferents, and thus that non-directional adaptation of central mechanisms is likely to be responsible for the tSAE.
